# The Combat against National Deterioration

**Published:** 1910-07-23

**Authors:** 


### SPECIAL ARTICLES.

### THE COMBAT AGAINST NATIONAL DETERIORATION.

### I.—THE WORK OF THE LADY ALMONER.

Already the first steps are being taken in the voluntary hospitals towards the gigantic task of bringing health into the home. The vast opportunities, too long ignored, presented by the out-patient departments have at length fallen under the observation of the social reformer, and it is inspiring to find that the impulse has come from within the institutions rather than from without, for it vindicates the voluntary hospital system, and opens up an almost limitless field of personal service when the old-fashioned out-patients' department shall have become a thing of the past.

#### What the Almoner has Done.

It is the lady almoner who has brought light and hope into the perplexing problem of the out-patient. She is at work now in nearly all the hospitals whose out-patient departments are thronged, and it was high time that she set about her duties, for she has laid bare many a lively abuse hitherto only dimly suspected. Workhouse inmates drifting round to the hospital for a bottle of stuff; energetic patients attending five or six different hospitals at the same time and apparently doing pretty well on the combined prescriptions; ladies with £800 a year bringing their children for special treatment; a large proportion (8 per cent. at St. Thomas's and a total of 367 cases at the London) proved well able to pay for their own doctor—these things show that the almoner cannot be dispensed with under existing conditions. But if this were all then the labours of these officials would be among the dreariest which the social order demands. Happily the hospitals were able to enlist the right kind of women for the visiting of their out-patients. Zeal, intellect, and tender charity combined have marked out a line of action through which many hopelessly despondent invalids are being restored to health and work.

#### At St. Thomas's Hospital: Maternity Work.

The report of the lady almoner for St. Thomas's Hospital deserves to be widely read and pondered over. Into it is packed such a lively faith in humanity, such proof of the regenerating force of personal service, as should make carping critics start forthwith on the task of helping in place of depreciating. The work carried out under the direction of Miss Cummins, the lady almoner, falls under two main headings: the Maternity Department, for which Miss Richmond is responsible, and the General Almoner's Department, in which the prevention of tuberculosis is a marked feature.

The main feature of the maternity work done by Miss Richmond and her voluntary assistants is the encouragement of thrift, which in the case of an expected confinement is a symptom of something far more valuable than the mere saving instinct. Thrift stands for preparation, for a right appreciation of the demands made upon father and mother by parentage, and for some intelligent attempt to meet their duties. Miss Richmond writes: "8,546 visits have been paid, and nearly a thousand babies have been watched during the year. Terms of personal friendship are constantly established between visitors and patients. The baby is looked on as a joint responsibility . . . it is very satisfactory to note an increase in the number of women who have either paid into a savings-bank, or tried to save at home for the nourishment necessary at the time of confinement, and also the preparations have been adequate in very many more cases. . ."

All this is spade work, but it is likely to have good results in the future, so that "the girls as they grow up and marry will have a good house tradition to go on in the management of their children."

#### Out-Patient Work.

Excellent as the maternity work is, it happily has its parallel in many localities where health visitors are bringing members of their own sex help and instruction at the time when it is most needed. Miss Cummins' labours in connection with the out-patients' department are more complex, and the rules which guide her in her treatment of individuals have all to be evolved for the occasion. The aim is "to ensure as far as possible that all out-patients to whom treatment is granted shall benefit to the full by that treatment. With this object, neither time nor labour are spared to secure, with the assistance of outside charitable agencies if necessary, the full co-operation of the patient in carrying out the treatment prescribed by the hospital." In the effort to carry out this aim it is discovered that ordinary hospital treatment is more often than not simply pouring water into a sieve. As for instance, "the rickety child is dragged to and from the hospital weekly, and the parents ignore the fact that the damp and dark basement in which it lives and sleeps is one of the causes of its condition . . . the parents have to be told that they must move, the local relief centres are approached, and . . . the charitable agencies are generally quite willing to co-operate in providing funds." The most teachable patients appear to be the phthisical, and Miss Cummins relates many cheering cases in which the tone of the whole family has been raised and a completely new start effected through the instruction and generously supported advice of the visitors. It is work of a kind which tests the workers, and at the same time trains them in knowledge of what extreme poverty involves. The good it does is handed on, and endures not for this generation only. A dozen people taught how to adjust themselves to town life so that they may preserve their health amid circumstances of difficulty, with heredity possibly against them and no money for extras, are a better witness to the power of the voluntary hospital than a hundred successfully treated for diseases which might have been prevented and will probably recur.

The pity of it is that the very thoroughness of the system admits of its benefits being extended to but few out of the sufferers who present themselves at the out-patients' department. The most pressing need seems to be for better provision for the feeble-minded and epileptic. These, not in their own interests alone, require segregation, and the task of providing suitable accommodation for them is only at the beginning. Extreme poverty mars the hopes of many other patients, and unhealthy employments destroy slowly but surely a large proportion of others. In all these directions, however, the hospitals which courageously grapple with the sources of disease are doing work which tends to open the eyes of the public at large to the preventable character of that suffering which they are endeavouring to assuage. When this truth is once appreciated, the task of altering existing conditions will not long be delayed.

